# Combined Approach of Cyclodextrin Complexationand Nanostructured Lipid Carriers for the Development of a Pediatric Liquid Oral Dosage Form of Hydrochlorothiazide

**DOI:** 10.3390/pharmaceutics10040287

**Published:** 2018-12-19

**Authors:** Marzia Cirri, Francesca Maestrelli, Paola Mura, Carla Ghelardini, Lorenzo Di Cesare Mannelli

**Affiliations:** 1 Department of Chemistry, School of Human Health Sciences, University of Florence, Via Schiff 6, Sesto Fiorentino 50019 Florence, Italy; marzia.cirri@unifi.it (M.C.); paola.mura@unifi.it (P.M.); 2 Department of Neuroscience, Psychology, Drug Research and Child Health (NEUROFARBA), Pharmacology and Toxicology Section, University of Florence, Viale Pieraccini 6, 50139 Florence, Italy; carla.ghelardini@unifi.it (C.G.); lorenzo.mannelli@unifi.it (L.D.C.M.)

**Keywords:** hydrochlorothiazide, drug-in cyclodextrin-in nanostructured lipid carriers, oral liquid formulations for children, gastric stability, storage stability, in vivo diuretic effect

## Abstract

The development of specific and age-appropriate pediatric formulations is essential to assure that all children and their care-givers can easily access to safe and effective dosage forms. The need for developing specific pediatric medicinal products has been highlighted by the European Medicines Agency. The aim of this study was to investigate the effectiveness of combining the advantages of both cyclodextrin (CD) complexation and loading into nanostructured lipid carriers (NLC), to obtain a liquid oral pediatric formulation of hydrochlorothiazide (HCT), endowed with safety, dosage accuracy, good stability and therapeutic efficacy. Equimolar drug combinations as physical mixture (P.M.) or coground product (GR) with hydroxypropyl-β-cyclodextrin (HPβCD) or sulfobutylether-β-cyclodextrin (SBEβCD) were loaded into NLC, then characterized for particle size, homogeneity, Zeta potential, entrapment efficiency, gastric and storage stability. The presence of HPβCD allowed higher entrapment efficacy than NLC loaded with the plain drug, and enabled, in the case of GR systems a complete and sustained drug release, attributable to the wetting and solubilising properties of HPβCD toward HCT. In vivo studies on rats proved the superior therapeutic effectiveness of HCT-in HPβCD-in NLC formulations compared to the corresponding free HCT-loaded NLC, thus confirming the successfulness of the proposed approach in the development of an efficacious liquid oral formulation of the drug.

## 1. Introduction

The development of specific and appropriate pediatric formulations is essential to assure that all children, of different ages, as well as their care-givers, can easily access to safe and accurate dosage forms. The European Medicinal Agency recently pointed out the need for developing medicinal products appositely designed for use in pediatric population, by providing specific guidelines with harmonized recommendations on the different aspects to be taken into account [1]. In fact, the lack of an adequate availability of medicines appositely developed for pediatric use resulted in the last decades in several off-label and unlicensed prescriptions, increasing the risks of adverse drug reactions, which may be more severe or different from those in adults [2]. Moreover, this problem also had as a consequence a wide use of extemporaneous preparations, obtained, starting from formulations for adult people, by dilution of liquid dosage forms or by tablet crushing or capsule opening, leading to administration of drugs with poor/uncontrolled dosing accuracy, unknown stability, variable bioavailability, poor compliance for children and care-givers [3]. Furthermore, the excipients of tablets or capsules for adults could be potentially toxic or unsuitable for pediatric patients [4]. The obtainment of a well homogeneous distribution of the drug is an additional issue to be taken into account, due to its very low dosage.

Diuretic drugs are often used in hypertension treatment of pediatric patients and, among these, hydrochlorothiazide (HCT) is included in the WHO Model List of Essential Medicines for children [5]. Extemporaneous liquid pediatric formulations of HCT are generally prepared in hospital pharmacies by dispersion of the drug powder in a sugar solution [6] or by suspension in a liquid vehicle of a ground fraction of a commercial tablet [7], with all the drawbacks above described [3]. In addition, it must be considered that HCT, a class IV drug according to the Biopharmaceutic Classification System (BCS) [8], presents problems of limited and variable bioavailability, related to its low solubility and permeability [9,10]. Moreover, the poor solubility and limited stability of HCT in aqueous solution [11], make the development of stable aqueous liquid formulations of the drug very difficult [12,13]. In fact, at present, there are not liquid formulations of HCT available on the market, while they should be very useful, particularly in the pediatric field.

The use of liquid lipid-based nanocarriers is a promising and versatile approach for delivery of hydrophobic drugs, due to their safety, biocompatibility and biodegradability, and their ability to improve dissolution and permeation properties of drugs, thus enhancing their oral bioavailability [14,15]. Among these, solid lipid nanoparticles (SLN), a colloidal carrier class composed by solid, physiologically compatible lipids dispersed in an aqueous surfactant phase, represent an interesting alternative to other colloidal systems such as liposomes or microemulsions; in fact, they offer analogous benefits, but allow to overcome their principal drawbacks, such as the limited entrapment efficiency and poor stability [16,17]. Moreover, the presence of the solid lipid matrix provides further advantages, including the protection of the embedded drug and the possibility of obtaining a controlled release [18,19,20]. A HCT-in SLN oral pediatric formulation has been recently successfully developed, endowed with satisfying entrapment efficiency (around 57%) and 1 month stability at 4 °C [21]. Nanostructured lipid carriers (NLC) have been recently proposed as an improved generation of lipid nanoparticle, with the aim of overcoming some potential drawbacks shown by SLN, such as in particular the tendency to expel the drug during storage as a consequence of their highly ordered crystalline structure [22,23]. NLC are composed by a solid lipid matrix, consisting of a mixture of biocompatible solid and liquid lipids, and an aqueous phase containing a surfactant or a blends of surfactants. The presence of the liquid lipids leads to a special nanostructure, with a less-ordered crystal lattice and increased imperfections in the solid matrix core, thus allowing to maintain the advantages of SLNs as drug carriers, while offering some additional important benefits such as facilitated incorporation of higher drug amounts, enhanced physical stability, lower drug leaking during storage [24,25]. Recently, NLC have been investigated to improve the oral bioavailability of different poorly water-soluble drugs [26,27].

A HCT-in NLC oral pediatric formulation has been recently successfully developed [28], which exhibited improved drug entrapment efficiency and higher stability under storage with respect to the corresponding previous SLN formulation [21].

Cyclodextrin (CD) complexation has been widely and successfully used to enhance solubility, dissolution rate and thus the bioavailability of several poorly soluble drugs [29] including HCT [30,31].

The effectiveness of simultaneously exploiting the advantages of both CD and nano-lipid carriers by joining them in a unique drug delivery system, by loading the drug-CD system into the lipid nanoparticles, has recently been reported [32,33,34,35]. In particular, loading into SLN of HCT as HPβCD binary system rather than as plain drug allowed to enhance the entrapment efficiency from 57% up to 66%, to prolong the formulation stability at 4 °C from 1 to 3 months, and to improve the drug release, with an almost 3-times increase of the released amount after 6 h [21].

Therefore, based on all these considerations, we deemed it worthy of interest to investigate the usefulness of a combined strategy based on loading HCT as CD system into NLCs formulations, to obtain a stable and effective liquid formulation of HCT suitable for the oral pediatric therapy. With this purpose, the effect of HCT complexation with two different β-CD derivatives was considered, i.e., hydroxypropyl-β-CD and sulfobutylether-β-CD selected due to their absence of toxicity, being the only two β-CD derivatives admitted for parenteral use [36] and present in the FDA’s list of Inactive Pharmaceutical Ingredients. The effect of CDs was investigated in terms of particle size and homogeneity, Zeta-potential, entrapment efficiency, gastric and storage stabilities and drug release properties of NLCs, compared to the previously developed NLC formulations containing the plain drug. Finally, the best HCT-CD-loaded NLC formulation was chosen for performing in vivo studies on rats, in order to evaluate its actual effectiveness in enhancing diuresis with respect to the corresponding formulation loaded with the plain drug and to a simple drug aqueous suspension.

## 2. Materials and Methods

### 2.1. Materials

Hydrochlorothiazide (HCT) was kindly supplied by Menarini (L’Aquila, Italy). Hydroxypropyl-β-cyclodextrin (Kleptose^®^HP, HPβCD average substitution degree DS = 0.85) and β-cyclodextrin were a gift from Roquette (Lestrem, France) and Sulfobutylether-β-cyclodextrin (Captisol^®^, SBEβCD) from Ligand Pharmaceuticals, Inc. (San Diego, CA, USA). Precirol^®^ATO5 (Glyceryldistearate NF/glycerylpalmitostearate), was kindly provided by Gattefossé (Milan, Italy); polyoxyethylene (Tween^®^80) was from Merck (Hohenbrunn, Germany), Poloxamer 188 (Pluronic^®^F68) from BASF (Ludwigshafen, Germany) and castor oil from Galeno (Prato, Italy). Purified water was obtained by reverse osmosis (Elix^®^ Millipore, Baltimore, MD, USA). All other chemicals were of analytical grade.

### 2.2. Phase-Solubility Studies

Phase-solubility studies were performed at 25 °C adding an excess amount of drug (50 mg) in 10 mL of pH 5.5 phosphate buffer solutions, containing increasing concentrations of HPβCD or SBEβCD (0–25 mM) put in sealed glass containers, preserved from the light and electromagnetically stirred (500 rpm) [37]. Aliquots were withdrawn every 24 h with a filter syringe (0.45 μm pore size) until equilibrium (72 h), and spectrometrically assayed for drug content at 272.2 nm (UV–vis 1600 Shimadzu spectrophotometer, Tokyo, Japan). Each test was carried out in triplicate (coefficient of variation, C.V. < 4%). The apparent stability constant (K_1:1_) and the complexation efficiency (CE) of the complexes were estimated using the following equations:

(1) $$K_{1:1}=\frac{\mathrm{slope}}{S_{0}\left(1-\mathrm{slope}\right)}$$

(2) $$\mathrm{CE}=\frac{\mathrm{slope}}{1-\mathrm{slope}}$$

where the slope is calculated from of the straight line portions of the phase-solubility diagrams and S_0_ is the drug solubility in the absence of CD.

### 2.3. Preparation of Drug-CD Binary Systems 

Physical mixtures (P.M.) of HCT with HPβCD and SBEβCD at 1:1 drug to carrier molar ratio were obtained by simple blending the powders for 15 min in a turbula mixer. Coground products, (GR) were instead obtained by grinding physical mixtures in a high-energy vibrational micromill (Mixer Mill Type MM 200, Retsch, GmbH, Düsseldorf, Germany) at 24 Hz for 30 min according to a previously developed method [21]. HCT was submitted to the same process for comparison purpose.

### 2.4. Dissolution Rate Studies

Dissolution studies were carried out according to the dispersed amount method. A sample of 200 mg of drug (untreated or submitted to grinding) or drug-equivalent, previously sieved (75–150 μm granulometric fraction), was added to 75 mL of pH 5.5 phosphate buffer solution thermostated at 37 ± 0.5 °C, in a 150 mL beaker. A three-blade paddle (1.5 cm radius) was centrally put in the beaker and rotated at 100 rpm. Aliquots (3 mL) periodically withdrawn with a syringe-filter (pore size 0.45 μm) were spectrometrically assayed for drug content as described above, and replaced with an equal volume of fresh medium. A correction for the cumulative dilution was made. The results are the mean of four experiments.

### 2.5. Preparation and Characterization of NLC

NLC were prepared by the hot high-shear homogenization technique followed by ultrasonication [21].The aqueous phase, containing a nonionic hydrophilic surfactant (Pluronic^®^F68 or Tween^®^80, 1.5% *w*/*w*), was heated up to 65 °C (i.e., 10 °C above the melting point of the lipid mixture) and dispersed in the molten lipid phase (Precirol^®^ATO5) added of the liquid lipid (castor oil) by 2 min stirring at 10,000 rpm with a T25 digital ULTRA-TURRAX^®^ homogenizer (IKA^®^Werke, Staufen, Germany). The obtained pre-emulsion was then sonicated for 3 min by a Sonopuls HD 2200 sonicator (Bandelin Electronics, Berlin, Germany) and finally cooled at 4 °C to solidify the lipid matrix and produce NLC.

Drug-loaded NLC were prepared by incorporating HCT as such (200 mg) in the lipid phase or by adding the selected HCT-CDsystem (as P.M. or GR, equivalent to 200 mg HCT) to the aqueous surfactant solution, respectively. The final drug concentration was 0.2% *w*/*v*.

All samples were protected from light and stored at a temperature of 4 °C.

### 2.6. Physicochemical Characterization of NLC

Particle size, Polydispersity Index (PDI) and Zeta-potential of the different HCT-loaded NLC formulations were determined by dynamic light scattering (DLS) using a Zetasizer Nano-ZS90 (Malvern Instruments Ltd., Worcestershire, UK) after appropriate dilution with purified water in order to prevent possible multiscattering phenomena.

Entrapment Efficiency (EE%) and loading capacity (LC%) of HCT-loaded NLC were determined indirectly, by determining the concentration of free drug present in the aqueous phase after ultrafiltration of the colloidal dispersion using a membrane concentrator (Vivaspin^®^2, 10,000 kDa MWCO, Sartorius Stedim Biotech Ltd., Göttingen, Germany). An aliquot (1.5 mL) of HCT-loaded NLC aqueous dispersion was placed in the upper chamber and centrifuged 8 min at 4000 rpm (HERMLE Labortechnik, mod. Z200A, Wehingen, Germany). The un-encapsulated drug, collected in the filtrate recovered in the lower chamber, was spectrophotometrically assayed at 272.2 nm (UV-1601 spectrophotometer, Shimadzu Corporation, Kyoto, Japan). No interferences by lipids and other components were detected in the spectrophotometric determination of the drug.

The following Equations (3) and (4) were used to calculate entrapment efficiency (EE%) and loading capacity (LC%), respectively:

(3) $$\mathrm{EE}\%=\frac{W_{\mathrm{initial}\;\mathrm{drug}}-W_{\mathrm{free}\;\mathrm{drug}}}{W_{\mathrm{initial}\;\mathrm{drug}}}\;\times \;100$$

(4) $$\mathrm{LC}\%=\frac{W_{\mathrm{initial}\;\mathrm{drug}}-W_{\mathrm{free}\;\mathrm{drug}}}{W_{\mathrm{lipid}}}\times 100$$

where W_init__ial drug_ is the total amount of drug initially added, W_f__ree drug_ is the amount of un-encapsulated drug and W_lipid_ is the weight of the total lipids used.

For each sample five separate experiments were carried out and the results are reported as the mean values ± S.D.

### 2.7. Stability Studies Under Gastric Conditions

The intra-gastric stability of empty NLC and NLC loaded with HCT alone or in combination with CD was evaluated in a simulated gastric solution (pH 4.5 phosphate buffer, miming the infant gastric pH [38]. Samples of 1 mL of each dispersion were suspended in 100 mL of simulated gastric solution thermostated at 37 °C. Size and PDI of NLC dispersions were measured immediately (t_0_) and after 2 h incubation (t_2 h_). Experiments were performed in triplicate and the results represent the average values ± S.D.

### 2.8. Storage Stability Studies

NLC formulations were stored at 4 °C for three months. At given time intervals (1 month), the mean particle size, PDI and Zeta Potential were determined in triplicate and the results were averaged. Possible phenomena of crystallization, formation of precipitates, gelling and/or growth of moulds were also checked by visual inspection.

### 2.9. Drug Release Studies

In vitro HCT release experiments from NLC formulations were carried out in sink conditions, by the dialysis bag method [32].The results were compared to those obtained from 0.2% *w*/*v* aqueous suspensions of the drug, alone or as equimolar P.M. or GR system with CD. 1 mL sample of NLC dispersion or drug aqueous suspension (all containing 2 mg of drug) was put into a cellulose acetate dialysis bag (Sigma-Aldrich, St. Louis, MO, USA, 12,500 cut-off) previously soaked overnight in pH 4.5 phosphate buffer (miming the infant gastric pH) and sealed with a clamp at both sides to prevent leakage. The bag was placed 2 h in 100 mL of pH 4.5 gastric buffer (HCT solubility in the medium about 0.7 mg/mL) and then 4 h in 100 mL of pH 6.8 phosphate buffer (simulating the intestinal pH), both thermostated at 37 °C and stirred at 50 rpm. 

The HCT released amount was spectrometrically assayed at 272.2 nm at predetermined times. After sampling of the receiver solution, an equal volume of fresh solvent was replaced, to keep the volume constant. A correction for the cumulative dilution was made. Experiments were carried out in triplicate and the results are expressed as the mean values ± S.D.

### 2.10. In Vivo Studies

Male Sprague-Dawley rats (Harlan, Varese, Italy) weighing around 200–250 g at the start of the procedure, were used for all the in vivo studies. The rats, housed in CeSAL (Centro Stabulazione Animali da Laboratorio, University of Florence, Italy), were used not before a week by their arrival. Animals, housed in suitable cages (4 for cage of 26 × 41 cm), were acclimatized at a temperature of 23 ± 1 °C, subjected to a 12 h light/dark cycle, (with light at 7 a.m.), and fed with a standard laboratory diet and tap water ad libitum. All manipulations of animals were performed in agreement with the Directive 2010/63/EU of the European Parliament and of the European Union Council (09/02/2010) on the protection of animals used for scientific aim. The ethical policy of Florence University complies with the Guide for Care and Use of Laboratory Animals of US National Institutes of Health (NIH Publication No. 85–23, revised 1996; University of Florence assurance number: A5278-01). Formal approval to carry out the experiments (project 108678, 28/06/2017) was given by the Animal Subjects Review Board of the University of Florence. Experiments involving animals have been carried out in compliance with ARRIVE guidelines [39]. Every effort has been made to minimize animal suffering and restrict the number of animals to use. 

The diuretic activity was determined following the method of Compaore et al. [40] with some variations. Animals were randomly divided into five groups (n = 5) for the acute (single dose) study with free access to food and water. Before each treatment, the rats received an oral dose of 2.5 mL/100 g body weight of physiological saline (0.9% NaCl) to impose a uniform water and salt load [41]. One hour later, a dose of 10 mg kg^−1^ was orally administered to the rats in the following manner: control group, with 0.9% saline; group I with empty NLC; group II with HCT aqueous suspension (0.2% *p*/*v* HCT); groups III and IV with the selected NLC formulation loaded, respectively, with the drug alone or as GR with HPβCD. Immediately after administration, the rats were put in suitable metabolic cages (one rat per cage), specifically designed to separate urine from feces. The cumulative urine output at 1, 2, 4, 6 and 24 h was determined. The diuretic activity was expressed as mL/100 g of body weight [42]. The diuretic index was obtained by the ratio of the diuresis of rats treated with the test formulation and that of the control group [43]. AUC_0–6 h_ and AUC_0–24 h_ (area under the urine volume curve versus time) were calculated according to equation (5):

(5) $$\mathrm{AUC}=\frac{1}{2}\left(V_{1}+V_{2}\right)\left(t_{2}-t_{1}\right).$$

where V is the volume of urine (mL) recovered at the time t_(h)_.

### 2.11. Statistical Analysis

One-way analysis of variance (ANOVA) followed by the Student-Newman-Keuls multiple comparison post test was employed to statistically analyse the results of all in vitro experiments (Graph Pad Prism version 6.0 software, San Diego, CA, USA). The results of in vivo studies were treated by one-way analysis of variance, applying a Bonferroni’s significant difference procedure as a post hoc comparison (Origin 9 software, OriginLab, Northampton, MA, USA). In all cases, the differences were deemed statistically significant when *P* < 0.05 or <0.01.

## 3. Results and discussion

### 3.1. Phase Solubility Studies 

Phase-solubility studies evidenced the solubilizing and complexing properties of the examined CDs towards HCT. A_L_ type phase-solubility diagrams were obtained with both CDs, characterized by a linearincrease of drug solubility with increasing CD concentration, as a consequence of the formation of soluble complexes [37] (see the Supplement, Figure S1). The 1:1 apparent stability constants (K1:1) of HCT-HPβCD and HCT-SBEβCD complexes were 106 and 210 M^−1^, respectively and the calculated complexation efficiency (CE) values were 0.220 and 0.436, respectively.

### 3.2. Dissolution Studies 

**Figure 1:**
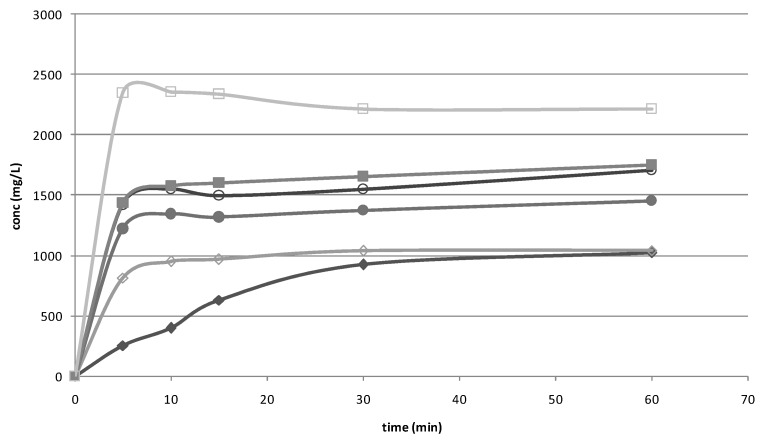
Dissolution rate studies performed with HCT alone (◆), HCT ground (◊) and the binary systems with HPβCD (P.M. ●; GR ○) and SBEβCD (P.M. ■; GR □).

Dissolution studies were performed on the drug alone, untreated or submitted to grinding procedure and on the drug CD P.M. and GR systems. As showed in Figure 1, ground HCT showed an initial faster dissolution rate than the untreated drug, due to the particle reduction induced by the treatment. The P.M. obtained with both CDs showed a more evident increase in drug dissolution rate, attributable to the wetting and solubilising effect of the CDs; this effect became even more marked for GR products, in virtue of the better interaction established between drug and CD by the co-grinding treatment. Previous solid state studies performed by DSC and X-ray powder diffractometry showed total drug amorphization and/or complexation in its GR products with HPβCD and HCT-SBEβCD [21].According to the results of phase solubility studies, SBEβCD demonstrated higher solubilising power towards HCT with respect to HPβCD.

### 3.3. Preparation and Characterization of Drug-Loaded NLC

In a previous study, we successfully developed a liquid pediatric oral NLC formulation of HCT [28], which showed better properties in terms of stability and entrapment efficiency than the previously developed SLN formulation of the same drug [21].

Therefore, as a continuation of this study, in the present work we investigated the possibility of further improving the effectiveness of HCT as NLC formulation, by loading the drug as CD system, considering the positive results obtained by such a combined approach in the case of SLN formulations [21]. With this aim, new series of NLC formulations composed by Precirol^®^ATO5 and castor oil (90:10 *w*/*w* ratio) as solid and liquid lipid, respectively, were prepared. Tween^®^80 or Pluronic^®^F68 (1.5% *w*/*w*) were used as surfactantssince previously selected as the most suitable ones [21,28]. The formulations were loaded with the drug as such (reference formulation) or in combination (in a 1:1 molar ratio) with selected CDs (HP^®^CDor SBEβCD), as simple physical mixture (P.M.) or as coground product (GR) [21]. To be able to evaluate the effect of the CD presence on NLC formulations compared to that previously observed for the corresponding SLN formulations, the same hot high-share homogenization method followed by ultrasonication was used for nanoparticle preparation.NLC formulations were characterized in terms of mean particle size, Polydispersity Index, Zeta-potential, Encapsulation Efficiency and Loading Capacity (see Table 1).

As previously observed in the case of SLN dispersions [21] and in accordance with the findings of other authors [44,45], Tween^®^80 gave rise to nanoparticle of larger size than Pluronic^®^F68, probably as a consequence of its different affinity for lipids that give rise to differences in incorporation of the surfactant in the outer shell of the particles [46]. In fact, Tween^®^80 due its lower HLB (Hydrophilic lipophilic balance) with respect to Pluronic^®^F68 (15 vs. 29 as reported in the technical sheets) can better interact with the lipid components of NLC, giving rise to a larger thickness of the coating layer on the particle surface and, consequently a greater particle size.

Loading of the drug in combination with HPβCD did not negatively influence NLC formation, whose mean particle size resulted only slightly lower with respect to that of the corresponding NLC formulations loaded with the free drug, regardless the type of surfactant used. The low PDI values, similar to those of free HCT-loaded NLC, suggested the obtainment of uniformly nano-dispersed colloidal systems. Negative Zeta potential values, all higher than 30 mV, were obtained, almost unchanged with respect to free HCT-loaded NLC. No important differences were observed when adding the drug as P.M. or as GR product with HPβCD. All these results indicated that the presence of HPβCD itself did not affect the physicochemical properties and stability of NLC dispersions. 

On the contrary, the addition of HCT in combination with SBEβCD, in the presence of both Pluronic^®^F68 or Tween^®^80 as surfactant, produced particles of large and not homogeneous dimensions, greater than 1000 nm and with PDI values higher than 1. Such a finding, analogous to that previously observed in the case of SLN dispersions [21] has been attributed to a possible interaction between the anionic SBEβCD and the surfactants present in both SLN and NLC formulations, leading to a variation in their solubilising capacity, thus modifying the water uptake by the lipid phase and, consequently, adversely affecting the particle formation and their stability [47].

Loading of HCT as equimolar system with HPβCD resulted in an increase of EE% with respect to NLC loaded with the plain drug, ranging from 11% in the presence of Tween^®^80 up to 70% in the presence of Pluronic^®^F68. Also in this case, no appreciable variations where detected when adding the drug-HPβCD system as simple P.M. or as GR product. Comparable positive effects on EE% of drug loading in combination with a suitable CD have been reported in case of SLN loaded with hydrocortisone as βCD complex [48] and of NLC loaded with ketoprofen as complex with polymeric βCD [32] obtaining an EE% increase of 70 and 77%, respectively, compared to the corresponding formulations loaded with the plain drug. A similar finding was also observed for SLN formulations of HCT in the presence of HPβCD [21]. Moreover, the EE% of the presently developed “drug-in HPβCD-in NLC” system was clearly higher than that of the previous “drug-in HPβCD-in SLN”. In fact, the best NLC formulation reached an EE% value nearly 89% vs. the 66.5% value obtained with the best SLN [21]. Analogous results were found for LC% values. The better drug loading ability of NLC with respect to the corresponding SLN formulations can be attributed to the formation of imperfections in the crystal lattice of the solid lipid (Precirol^®^ATO), due to the incorporation of the liquid lipid (castor oil), thus leading to a less ordered structure, able to accommodate larger drug amounts [49,50,51].

### 3.4. Stability Studies of NLC Formulations in Gastric Conditions 

The gastrointestinal stability of lipid formulations plays a crucial role to assure a controlled and reproducible drug release profile during their transit. In particular, the acidic medium may destabilize lipid carriers, thus leading to aggregation phenomena and, consequently, negatively influencing the drug release properties. The gastric stability of NLC dispersions was evaluated by determining any variation in the mean size and PDI of the particles after incubation at 37 °C for 2 h in pH 4.5 buffer solution, chosen as an average pH value for infant population [38].The addition of proteolytic enzymes, such as pepsin or trypsin, was not judged necessary, considering the lipid nature of the formulations under study. On the other hand, the use of a lipolysis model was not possible, since the differences between adults and pediatric population make the adaptability of the various in vitro models designed for adults unsuitable for miming pediatric conditions. In addition, it has to be considered the lack of commercial availability of Human Gastric Lipase (HGL) and Pancreatic Lipase-Related Protein 2 (PLRP2), reported as the most active lipolytic enzymes in neonates [52,53,54], as well as the difficulty of determining their levels [55].

**Figure 2:**
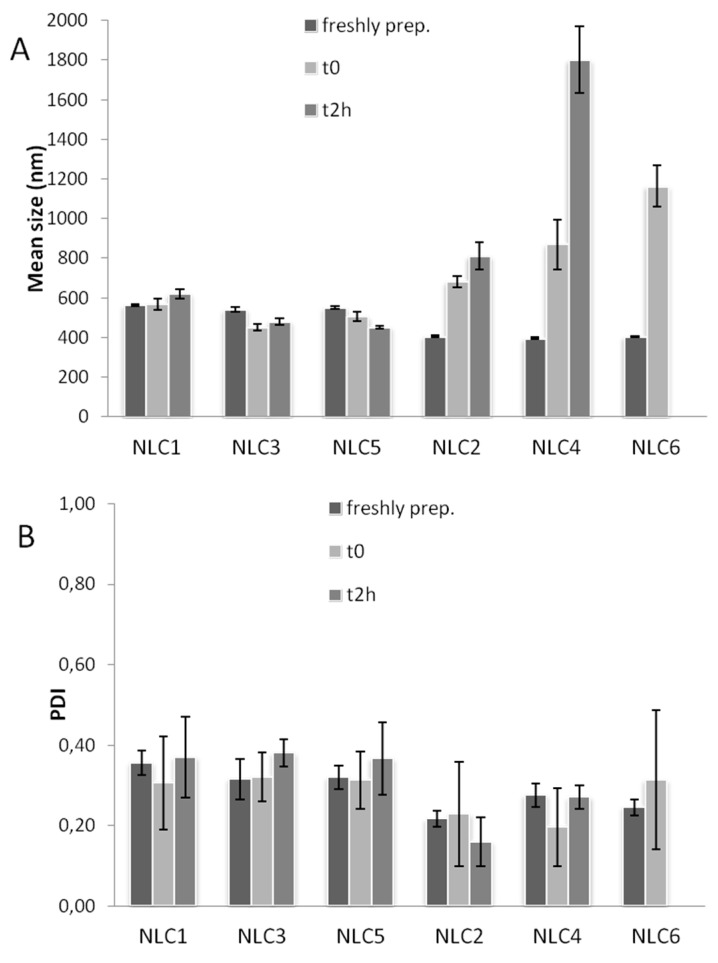
Gastric stability of Tween^®^80-based NLC (odd series) or Pluronic^®^F68-based NLC (even series) in terms of mean size (**A**) and PDI (**B**).

Figure 2 reports the gastric stability of the developed NLC formulations in terms of mean dimensions (A) and PDI (B).

The particle size of the Tween^®^80-based NLC (odd series) did not considerably change after 2h exposition, as previously observed for the corresponding SLN [21].

On the contrary, in the case of Pluronic^®^F68-based NLC (even series) a growth in the particle size was observed immediately after exposition to the gastric medium, and a further growth was found at the end of the test (2 h), indicative of strong aggregation phenomena induced by the low pH of the gastric medium. 

The different behaviour of NLC containing Pluronic^®^F68 with respect to the corresponding ones containing Tween^®^80, could be attributed to the different HLB values of the two surfactants that, having a different affinity for the lipids of NLC, as well for the gastric medium, give rise to a different stabilizing effect, as also observed by different authors [46,56,57,58]. Moreover, in the case of Tween^®^80-based NLC, an additional stabilizing effect was provided by HPβCD, probably attributable to interactions between Tween^®^80 and CDs enabling a synergistic interfacial tension decrease and viscosity increase of the dispersed system [59].

On the contrary, a concomitant destabilizing effect was revealed by Pluronic^®^F68-based NLC in the presence of HPβCD. It could be attributed to a possible interaction between the hydroxyl groups of HPβCD and the polypropylene oxide (PPO) blocks of Pluronic, leading to supramolecular structures, as reported by several authors [60,61,62]. Such an interaction, promoted by the low gastric pH, could lead to a decrease in the Pluronic^®^F68 surfactant function and, consequently, to a deconstrution of the lipid dispersions in the gastric environment. 

An analogous finding was previously observed for Pluronic^®^F68-based SLN formulations containing HPβCD [21].The low PDI values of NLC remained almost unchanged, thus indicating the maintenance of a homogeneous dimensional distribution for all the colloidal dispersions (Figure 2B).

### 3.5. Release Studies from NLC

**Figure 3:**
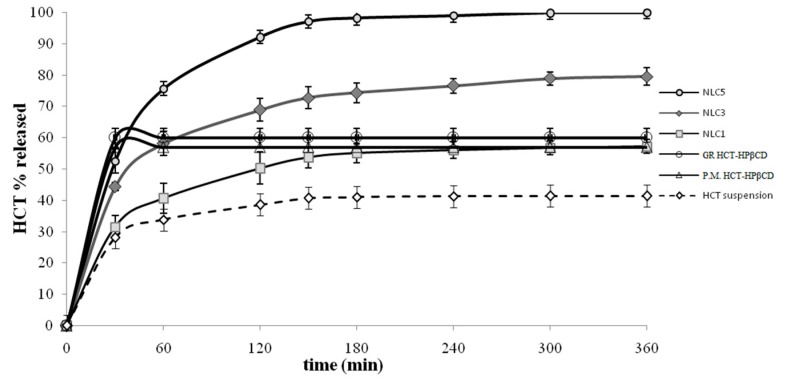
In vitro Hydrochlorothiazide (HCT) release profiles from Tween^®^80-based NLC containing the drug as such (NLC1) or as P.M. (NLC3) or GR system with HPβCd (NLC5), from its suspensions containing the drug as such or as P.M. or GR systems with HPβCD, all at the same drug concentration (0.2% *w*/*v*).

Based on the results of gastric stability studies, Pluronic^®^F68-based NLC were then discarded, whereas the series of Tween^®^80-based NLC was selected for in vitro release studies. The release profiles of these formulations loaded with the drug as such or as P.M. or GR system with HPβCD are depicted in Figure 3, together with that of HCT-HPβCD GR or HCT aqueous suspension as reference, all at the same drug concentration (0.2% *w*/*v*).

The drug suspension exhibited an initial rather fast release rate, in virtue of the rapid diffusion of the drug fraction already present in solution; however, a plateau phase was rapidly achieved, slightly exceeding 40% released drug. A better drug release rate was observed for the NLC formulation loaded with the plain HCT (NLC1), which reached almost 60% released drug. Similarly, a greater drug release rate from its NLC formulation than from its suspension was observed also in the case of tacrolimus [63]. As expected, a faster drug release was obtained for P.M. and GR systems with HPβCD, which both reached the maximum released % (around 60%) after only 30 min. However, the total amount of released drug, even if higher than that of drug alone (around 40%) was incomplete, and similar to that obtained from NLC loaded with the plain drug (60% released after 6 h). On the contrary, significantly better release profiles were obtained from NLC containing HCT as P.M. (NLC3) (80% released after 6 h) and even more as GR (NLC5) (100% released after 6 h) with HPβCD. Such an improvement could be ascribed to the high wetting ability and solubilizing properties of HPβCD toward the drug combined with the effect of the nanocarrier. The initial fast release rate observed from all the NLC formulations could be attributed to the quick diffusion of unentrapped HCT. The better performance of NLC loaded with the coground product with respect to the simple physical mixture can be attributed to the more intense drug-CD interactions obtained in such system by the preparation method, and then to a more effective solubilizing effect. An analogous result was previously observed in the case of NLC dispersions loaded with ketoprofen as P.M. or GR product with a polymeric βCD derivative [32].A similar trend in the drug release rate (drug-in CD-in NLC > drug in NLC > drug suspension) has been reported in the case of vinpocetine [33].

### 3.6. In Vivo Studies

**Figure 4:**
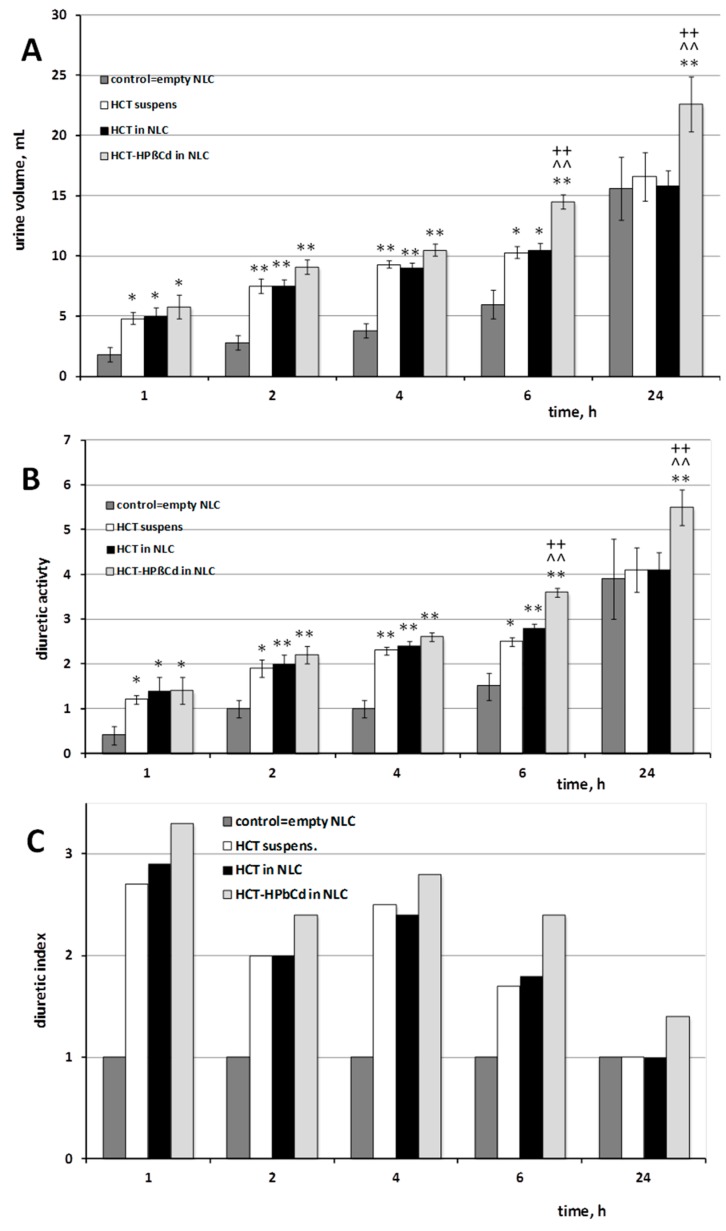
Diuretic activity in rats, expressed as volume of excreted urine (**A**), ml/100 g body weight (**B**) and diuretic index (**C**) after oral administration (10 mg kg^−1^) of Hydrochlorothiazide (HCT) as aqueous suspension or as GR product with HPβCD loaded into Tween^®^80-based NLC, compared to control group treated with a same volume of physiological saline (0.9% NaCl). * *P* < 0.05 and ** *P* < 0.01 vs. control group; ^^ *P* < 0.01 vs. HCT suspension; ++ *P* < 0.01 vs. HCT-loaded NLC.

The diuretic effect of theTween^®^80-based NLC formulation loaded with the drug as GR product with HPβCD, elected as the best formulation based on the results of in vitro release studies, was evaluated in rats, in comparison with that of the corresponding empty NLC, NLC formulation loaded with the plain drug and a simple drug aqueous suspension, all orally administered at the same dose (10 mg/Kg). The induced diuretic effect was measured as total volume of excreted urine, diuretic activity (volume of excreted urine per 100 g body weight), and diuretic index, with respect to a control group treated with saline (Figure 4).

Empty NLC showed similar values (*P* >> 0.05) to those obtained from the control in terms of both volume of excreted urine and diuretic activity, allowing to rule out any possible effect on diuresis exerted by the components of the nanoparticles.

Administration of the drug aqueous suspension produced a significant increase (*P* < 0.05) of the diuresis at 1 h after the treatment with respect to the control group, which was maintained up to 6h. Treatment with the drug as NLC dispersion showed a urinary output comparable to that induced by HCT suspension. An appreciable increase in the excreted urine volume and diuretic activity was instead observed in the case of administration of NLC dispersion loaded with the drug as coground product with HPβCD, which gave rise to higher values than those of both HCT suspension and plain HCT in NLC just after 2 and 4 h and displayed significantly (*P* < 0.01) superior effects starting from 6 h after treatment, which were maintained up to 24 h (Figure 4A,B). Similar results were observed also for the diuretic index (Figure 4C), confirming the superior therapeutic effectiveness of the HCT-in HPβCD-in NLC formulation with respect to the corresponding formulation loaded with the free drug. In virtue of the absence of diuretic effect shown by empty NLC, we can state that the combined approach of CD presence and entrapment into nanoparticles actually allowed an improved drug bioavailability leading to a superior therapeutic activity.

To better evidence the overall effect of the different treatments, the AUC_0–6 h_ and AUC_0–24 h_ (area under the urine volume curve versus time) were determined. The data confirmed the improved bioavailability of HCT when administered as HPβCD complex loaded in NLC: in fact, the AUC_0–6 h_ and AUC_0–24 h_ values of such formulation were, respectively, about 25 and 40% higher than those of both the corresponding NLC formulations containing the plain drug and the drug suspension.

The better performance of the HCT-in HPβCD-in NLC formulation with respect to the corresponding NLC formulation loaded with plain HCT could be attributed to several factors, including the higher drug entrapment efficiency and, in particular, the better drug release profile; moreover, a possible permeation enhancer effect due to the CD presence could also be hypothesized. 

An improved bioavailability of NLC loaded with the drug as CD complex with respect to those containing the free drug has been reported in the case of Vinpocetine, and attributed to a solubilizing effect and a faster release rate of the drug from the lipid matrix, in virtue of the drug complexation [33].

On the other hand, a comparison with the in vivo performance of the corresponding HCT-in HPβCD-in SLN formulation was not possible, since it was discarded for in vivo studies; in fact, its too fast release rate shown by in vitro studies was considered unsuitable for obtaining the desired controlled release profile [21].

### 3.7. Stability Studies on Storage

**Figure 5:**
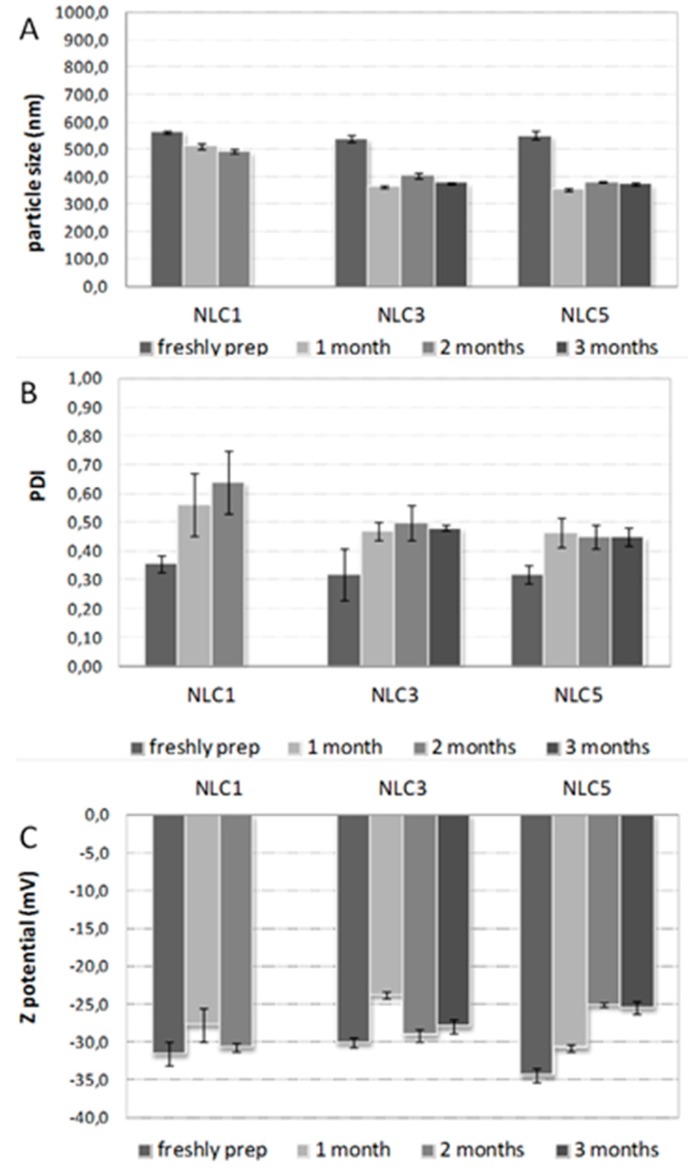
Stability studies of Tween^®^80-based NLC containing the drug as such (NLC1) or as P.M. (NLC3) or GR system with HPβCD (NLC5), freshly prepared and after 1, 2 and 3 months storage at 4°C in terms of mean size (**A**), polydispersity index, PDI (**B**) and Zeta potential (**C**).

NLC formulations were stored at 4 °C over 3 months and checked for mean dimensions, polydispersity index (PDI) and Zeta potential (Figure 5A–C). These experimental conditions were selected according to those used in the case of the previously developed NLC formulations loaded with the plain drug [21], in order to be able to compare the obtained results.

A moderate reduction of particle size was registered over the storage time, as previously observed in the case of SLN formulations [21]. It could be a consequence of some minor loss of lipids occurring in the presence of partially esterified glycerides such as Precirol^®^ATO5 [45,46]. NLC loaded with the pure drug were considered stable only up to 2 months, since the progressively marked increase in the PDI occurring over this time could indicate a very polydisperse system (Figure 5B). However, such formulation was more stable than the corresponding SLN, thus confirming the actual advantages of using NLC rather than SLN. On the contrary, no important variations of PDI values were observed for NLC3 and NLC5 containing the drug as CD system during all the examined period, indicating the existing of monodisperse homogeneous systems even after three months. Zeta potential kept negative values greater than −25mV during the whole storage time (Figure 5C), thus confirming the physicochemical stability of such formulations and the positive influence of the presence of the cyclodextrin, as previously observed [21].

## 4. Conclusions

In this study, the usefulness of a combined strategy, based on the drug interaction with a suitable CD, and the loading of the binary systems into NLC, has been evaluated in the development of an oral liquid formulation of HCT intended for pediatric use, able to assure safety, dosage accuracy, stability and therapeutic efficacy.Thus, the grinding procedure confirmed its efficiency for preparing cyclodextrin inclusion complex in the solid state [64].

Among the CDs initially examined as complexing agents for the drug, SBEβCDwas rejected, since its presence did not enable the formation of NLC of suitable nanometric dimensions. Instead, the addition of the HCT-HPβCDcombination, both as simple P.M. or as GR product, allowed the obtainment of homogeneous NLC dispersions, with mean particle size, PDI and Zeta potential values substantially similar to those of the corresponding NLC formulations loaded with the plain drug, but withsignificantlyincreased entrapment efficiency. 

Moreover, the presence of HPβCD, in virtue of its solubilizing and wetting properties, enabled to enhance the HCT release rate from NLC formulations with respect to those containing the plain drug. A more marked effect was foundin the case of NLC loaded with the HCT-HPβCDGR product, where a synergistic effect of two carriers allowed the obtainment of a complete and controlled drug release. In addition, the presence of CD positively influenced the stability of such formulations, due to its preservative effect.

Such last NLC formulation, selected for in vivo studies on rats, showed a significantly (*P* < 0.01) more intense and prolonged diuretic effect not only than a simple HCT aqueous suspension, taken as reference, but also than the corresponding NLC formulation loaded with the plain drug, definitely proving the actual successfulness of the proposed HCT-in HPβCD-in NLC formulative approach.

Further studies are ongoing, in order to investigate the possibility of extending the shelf-life of the developed formulation by freeze-drying the NLC dispersion and reconstitution at the time of use.

## Figures and Tables

**Table 1:** Composition of the different NLC formulations, all containing Precirol^®^ATO5 as solid lipid and castor oil as liquid lipid (90:10 *w*/*w* ratio) and their properties in terms of mean size, polydispersity index (PDI), Zeta potential (ζ), Encapsulation Efficiency (EE%) and Loading Capacity (LC%).

Formulation code	Surfactant type (1.5%*w*/*w*)	Drug-CD system *	Size (nm)	PDI	ζ (mV)	EE%	LC%
NLC1	Tween^®^80	HCT alone	563.3±3.7	0.36±0.03	−31.6±1.6	80.0±0.2	3.2±0.01
NLC3	Tween^®^80	HCT-HPβCD P.M.	539.8±10.9	0.32±0.09	−30.1±0.6	88.8±0.1	3.5±0.01
NLC5	Tween^®^80	HCT-HPβCD GR	550.1±15.1	0.32±0.03	−34.4±1.0	87.7±0.1	3.5±0.01
NLC7	Tween^®^80	HCT-SBEβCD P.M.	>1000	-	-	-	-
NLC9	Tween^®^80	HCT-SBEβCD GR	>1000	-	-	-	-
NLC2	Pluronic^®^F68	HCT alone	405.4±3.8	0.22±0.02	−38.8±1.4	40.1±3.2	1.6±0.13
NLC4	Pluronic^®^F68	HCT-HPβCD P.M.	395.6±3.7	0.28±0.08	−34.0±1.1	68.7±1.3	2.7±0.05
NLC6	Pluronic^®^F68	HCT-HPβCD GR	403.8±3.8	0.25±0.03	−34.3±0.7	68.2±0.5	2.7±0.02
NLC8	Pluronic^®^F68	HCT-SBEβCD P.M.	>1000	-	-	-	-
NLC10	Pluronic^®^F68	HCT-SBEβCD GR	>1000	-	-	-	-

* all equivalent to 200 mg HCT.

## Supplementary Materials

The following are available online at http://www.mdpi.com/1999-4923/10/4/287/s1, Figure S1: Phase solubility studies of HCT with SBEβCD (◆) and with HPβCD (■).
