# Supplementary material for: Combined Approach of Cyclodextrin Complexationand Nanostructured Lipid Carriers for the Development of a Pediatric Liquid Oral Dosage Form of Hydrochlorothiazide
Source: Pharmaceutics. 2018 Dec 19;10(4):287. doi: 10.3390/pharmaceutics10040287 (PMC6321408; doi:10.3390/pharmaceutics10040287)
Supplement: Supplementary file 1 [file pharmaceutics-10-00287-s001.pdf]

pharmaceutics

Supplementary Materials: Combined Approach of Cyclodextrin Complexationand Nanostructured Lipid Carriers for the Development of a Pediatric Liquid Oral Dosage Form of Hydrochlorothiazide

Marzia Cirri, Francesca Maestrelli, Paola Mura, Carla Ghelardini and Lorenzo Di Cesare Mannelli

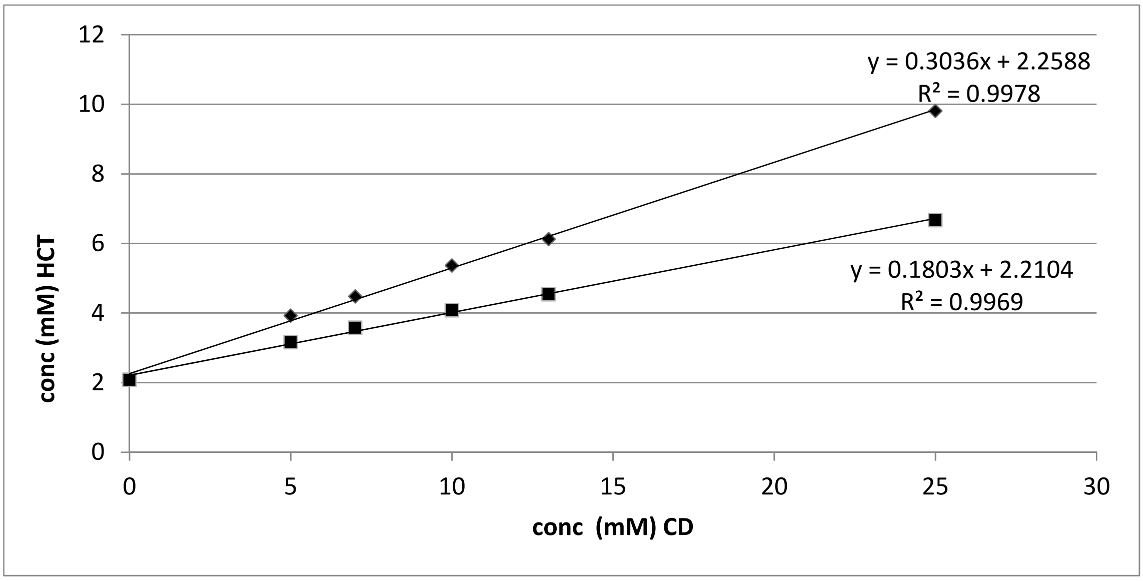

Figure S1. Phase solubility studies of HCT with SBEβCD (◆) and with HPβCD (■).
